# J. M. Rattray

**Published:** 1923-01

**Authors:** 


### Obituary.

### J. M. RATTRAY, M.A., M.D.

The death of Dr. John Moysey Rattray on November 4th, aged 65, causes a gap among our older medical colleagues; for although he had retired from his practice at Frome, he retained his untiring interest in all that concerned the profession he had served so well, and that cheerful, bright, conversational power that rendered him always a delightful companion, and made one forget that he was no longer young. Indeed, he was full of interests and work to the last evening before his sudden death from angina.

Bristol was deeply indebted to Rattray, for it was he who caused Greig Smith to come to us. When as recently-fledged fellow-graduates they were talking over prospects, what to do and where to set up in practice, perchance had it not been for Rattray's finger pointing to Bristol this *Journal*, so happily initiated by Greig Smith, might never have come into being.

J. M. Rattray was born in 1857, and was the son of Dr. John Rattray, a physician of Aberdeen. He graduated in medicine at Aberdeen University in 1878. He married a daughter of the late Canon Bennett, of Maddington, near Salisbury, and leaves a widow and two sons. He settled in Frome in 1881 as a partner of the late Dr. Cockey, subsequently remaining in practice there till he had passed his sixtieth year. He was a past President of the Bath and Bristol Branch of the British Medical Association.

A keen churchman and Conservative, he took an active share in the social and political life of his adopted town. He was an unusually widely-read and literary man, and both studied and wrote on many subjects, but especially devoted his attention to historical medicine after his retirement from practice, and in September, 1921, was elected a Fellow of the Royal Historical Society. He had made great progress in the study of the History of Medicine, and had he been spared would have doubtless published the results of some of his researches.

In 1917 he was shot by a patient who was out of his mind, resulting in a united fracture of the humerus and eventually his retirement from practice. To few is it given to meet such grave misfortune bravely and without bitterness, to show indomitable spirit and turn to fresh activities.

His elder son has been in practice for a long time in the Straits Settlements, and is now in Singapore.

To his widow, who threw herself into and so thoroughly shared in the manifold interests of their life at Frome and subsequently in his retirement in London, as well as to his family, we tender our heartfelt sympathy.

P. W. W.

